# The nature of youth in the eyes of mental-health care workers: therapists’ conceptualization of adolescents coming to therapy at others’ initiative

**DOI:** 10.1186/s13033-020-00363-w

**Published:** 2020-05-06

**Authors:** Tonje Børseth Barca, Christian Moltu, Marius Veseth, Gro Fjellheim, Signe Hjelen Stige

**Affiliations:** 1 Finnmark Hospital Trust, District Psychiatric Centre, Po.Box 1294, 9505 Alta, Norway; 2 Førde Hospital Trust, Førde, Norway; 3 University of Bergen, Bergen, Norway; 4 Bergen Hospital Trust, Bergen, Norway

**Keywords:** Adolescent, Therapy, Drop-out, Therapist view, Qualitative research, Focus groups, Interview

## Abstract

**Background:**

Adolescent clients often come to therapy at the initiative of others and show a higher drop-out rate compared to adult clients. Therapeutic relationships are critical for preventing drop-outs and attaining good outcomes, yet few empirical studies have investigated how therapists conceptualize and meet adolescent clients who come reluctantly to therapy.

**Methods:**

We conducted ten focus-group interviews in this study with a total of 51 therapists at different Norwegian specialist outpatient clinics for children and adolescents with mental-health problems to explore how therapists view and understand adolescents who come to therapy at the initiative of someone else. We used a reflexive approach to thematic analysis to analyze the transcripts.

**Results:**

We found five main themes, expressing variations in participants’ understanding: *The hurt and distrustful adolescent; The adolescent lacking hope for the future; The adolescent engulfed in the burden of mental*-*health suffering; The adolescent as something more than a psychiatric patient; and The adolescent meeting a system with varying flexibility and space for engagement.*

**Conclusions:**

Several conceptualizations of the adolescent client coexist within and between clinics, resulting in variability of services for adolescents even within the frames of a strong welfare system.

## Introduction

Clinical experience and some research indicate that a large portion of adolescent referrals to mental-health outpatient clinics are initiated by people other than the adolescent him/herself [1] such as parents, teachers, school nurses, or child-welfare services. Activity registries in Norwegian outpatient clinics for children and adolescents with mental-health problems have documented that many adolescents do not show up for their scheduled therapy sessions. Moreover, it is difficult to engage and keep adolescents in treatment, as demonstrated by a meta-analysis that estimated 28–75% of adolescents in mental-health care drop out of treatment [2]. In comparison, approximately 18–22% of all adults in treatment terminate it early; even among adults, however, younger age is associated with a greater likelihood of dropping out of treatment [3, 4].

Little is known about the consequences of these high dropout rates. Also, research exploring reasons for dropping out of treatment shows that there is a range of reasons for dropping out. Some adolescents drop out of therapy because they feel they have gotten what they needed from treatment [5]. This might in part explain why there is no strong evidence that adolescents dropping out from treatment have poorer clinical outcomes than completers [6]. Other clients may drop out of treatment because of dissatisfaction with therapy or the therapist, or because of more complex life situations [5]. The last two groups are important to keep in therapy. The scarce research that has explored therapist behavior in response to drop out suggest that therapists use personalized phone calls, letters or through staff or client´s family members to re-engage clients in treatment [7].

Important predictors of drop-out rates among adolescents include poorer therapeutic alliance early in treatment [8–11] and a decline in the quality of the therapeutic relationship during therapy [12]. The therapists are often unaware of the adolescents´ dissatisfaction [5]. Initial motivation is also an important predictor of treatment outcomes in adolescents [13–15]. The quality of the therapeutic relationship is essential for good outcomes with adolescents in psychotherapy [16], and therapists working with adolescents have to assume more responsibility and initiative to develop an alliance [17]. Qualitative studies and a meta-analysis of the variables affecting the therapeutic relationship both stress the importance of the therapist’s interpersonal competence, warmth, and empathy [18, 19].

In general, factors related to therapists and therapeutic relationships are widely acknowledged as being critical for preventing drop-outs and attaining good outcomes in psychotherapy [20–24]. Dropout is more dependent on the therapist treating the child, or which clinic the child attends to, than the characteristics of the case itself [25]. This knowledge points to the significance of understanding therapists’ views about both the therapeutic relationship and adolescent clients to understand therapeutic processes when adolescents come reluctantly to treatment.

Several factors influence therapists’ work with clients. Cognitive schemas and preconceptions influence how we interpret information and behave towards others and affect interpersonal interactions in general [26–28]. It follows that therapists’ interpretation of adolescents’ difficulties, lack of motivation, and resistance to therapy might affect the way they deal with adolescent clients and, consequently, affect motivation, risk of dropout, and therapeutic outcomes. In addition, therapy with adolescents is often similar to a challenging hill start, in that many adolescents are in therapy without seeing the point of being there. Research indicates that initial motivation and opportunities for alliance formation influence outcomes [29], which place particular responsibility on therapists when meeting adolescents who enter therapy at someone else’s initiative. In this paper, we explore the following research questions: How do therapists view adolescents who come reluctantly to therapy? and How do they conceptualize their own and the adolescent’s roles and responsibilities in treatment?

## Methods

### Study setting

This study was conducted in a specialized mental-health care setting for children and adolescents (0–18 years) in Norway. Norway has experienced exponential economic growth the past decades due to vast reserves of oil. Combined with a small population distributed across large areas and high acceptance of socialist ideas such as equal opportunities for all and redistribution of wealth, Norway has developed a strong welfare system. Norwegian culture is also characterized by low power distance [30] and a strong employment protection legislation, which means individual opinions and critical attitudes often are valued, also as a part of the clinical and professional autonomy. These are important contextual factors in understanding the presented research design and findings.

All treatment in specialized mental-health care for children and adolescents in Norway is free of charge. Admission happens after referral from primary healthcare and measures to improve the situation have been attempted. Health professionals in specialized mental health care include medical doctors, psychiatrists, clinical psychologists, psychiatric nurses, clinical special education teachers, and clinical social workers, and a high degree of interdisciplinarity is valued and desired. Adolescents between the ages of 12 and 16 are mandated to have partial shared-decision making regarding the healthcare they receive, and at 16 they are fully capable of autonomous consent concerning health issues. However, parents and other authorities often pressure adolescents to attend treatment. This makes the question of how to relate to adolescents who are not motivated to be in treatment particularly relevant.

This study was part of a larger project focusing on adolescents who did not initiate therapy themselves. The overarching project involved individual interviews with adolescents and focus groups with therapists. In the individual interviews we addressed the adolescents experience of coming to therapy, with paying particular attention to the initial phase of treatment and how this could affect their motivation for continued treatment. In the focus groups, on the other hand, the main focus was the therapists’ perspective on adolescents coming reluctantly to therapy, including their understanding why adolescents can be reluctant to enter therapy, and how therapists identified and worked therapeutically with adolescents entering therapy reluctantly. To do justice to the data material and fully explore the therapist view, this article presents data from the focus groups with therapists only. Analysis of the individual interviews with adolescent clients will be presented in a separate article.

### Design

Given the organization of specialized mental health care in Norway, including the high degree of interdiciplinarity, we chose focus-group methodology to explore therapists’ perspectives about adolescents who are reluctant to start therapy and who come to therapy at the initiative of others. Focus groups are well suited to elicit rich data—on content and interactions—about a group’s perspective on and experience with a phenomenon [31, 32]. In contrast to group interviews, where the interviewer takes more control of the interaction and has a more active role, we wanted to utilize the potential of the focus group to get a peek into a group’s interactions and communication in relation to the given topic, although the facilitators of course ensured minimal participation of all participants and facilitated the interaction when needed. To get a high naturalistic validity we chose to recruit already established teams and included their clinical leaders in the focus groups. Although involving a risk of participants feeling pressure and constraint due to their leaders being present, we assessed this to be the best option to explore our research questions, and justifiable ethically given the cultural context (see also data collection, ethics, and limitations sections for more information). By using focus groups instead of individual interviews with therapists, we would be able to get insight into the coexistence of different perspective, as well as how treatment teams relate to differences in perspectives. This would give us valuable information in relation to our research questions.

### Recruitment procedure and participants

The focus groups consisted of established teams of therapists working with children and adolescents. We contacted the management of outpatient clinics, who forwarded the invitations to participate in the project to their therapists on staff. Our aim was to recruit participants from different areas in Norway and from larger cities and rural areas. We recruited seven clinics in all, and conducted ten focus-group interviews, based on the organization of already established treatment teams within the participating clinics. Six clinics were general outpatient clinics for children and adolescents with mental-health problems. One clinic was more specialized, with one team working with adolescents developing early psychosis, and the other team using dialectical behaviour therapy with adolescents with self-harm problems and suicidal ideation. We recruited clinics from the western and northern parts of Norway.

The composition of each focus group reflected the naturalistic distribution of mental-health care workers in a specialized care setting in Norway with respect to professions (a high degree of interdisciplinary) and gender (the majority were women). The focus groups consisted of three to seven participants, with a total of 51 participants (40 women). The team leaders were present during all the focus-groups interviews, and the leader of the clinic was also present and participated in five focus groups. Participants had varied occupational and educational backgrounds (including clinical psychologists, psychiatrists, resident medical doctors, psychiatric nurses, clinical special education teachers, and clinical social workers), and ranged in age from the late 20 s to the late 50 s.

### Data collection

We conducted the focus-group interviews between November 2017 and January 2018. Data collection was conducted at the clinics during working hours to include as many therapists as possible. One or two researchers moderated each focus group. All the authors moderated at least one focus group. Two of the authors worked in clinics that were included in the study, but they did not participate in the interviews in their own clinics. Each interview lasted approximately 60 min. Since the participants were colleagues, they were already in established groups, and therefore, we were able to study the teams’ subculture and their ways of talking about and working with adolescents who come reluctantly to therapy, as well as exploring the perspectives and experiences of individual therapists.

We developed a semi-structured interview guide for the focus groups. The schedule opened with questions about the services provided by the clinic to get a basic sense of the organization of services and the team’s understanding of the work they do, and to build initial rapport with the group. Then, the discussion was directed more toward the interview’s main focus: i.e., the therapists’ experiences with and perspectives about adolescents who come to treatment reluctantly. We explored, in order: [1] whether they recognized the phenomenon; [2] how they understood this group of adolescents; and [3] what they did when they worked with adolescents who came to therapy reluctantly. The discussions were audio-recorded and transcribed verbatim for analysis. In addition, one of the moderators made notes to link quotes to the different participants, so we could differentiate the voices of different participants and get a picture of the interaction between participants from the transcribed material.

### Data analysis

We chose an explorative and reflexive thematic analysis and a team-based approach to explore the perspectives of the participants [33–35].

1. The analysis began by all the authors reading and re-reading the transcripts to familiarize themselves with the material and make notes about what they found interesting in it. We met for an analysis seminar where we discussed initial ideas and possible analytical foci. The analytic focus we decided on was: What is an adolescent in a mental-health care context?
2. The first author conducted the primary coding of the material to identify units of meaning across the dataset relevant to the analytical focus. NVivo 12 software [36] was used as technical support for analyzing the interview transcripts. All parts of the relevant text were examined and labeled with codes. The analysis was inductive, with the primary coding performed line-by-line and interview by interview.
3. The first, second, and last author, then went back and forth between the full interviews and the codes to identify parts and codes that belonged together, looking for broader patterns of meaning and possible themes across the dataset. They suggested three preliminary themes that described the present codes based on these patterns.
4. We then reviewed our three tentative themes by consulting the full data material to check whether relevant parts of the material were overlooked in the thematic structure and if the three themes covered the codes.
5. The first author refined the analysis by writing a tentative findings section with examples of quotes to explicate the thematic content. Then, sub-themes were identified to provide structure and to show a hierarchy of meaning in the data.
6. The first proposal of the three main themes and sub-themes were then sent to all the authors. Together, we modified the thematic structure and theme names, agreeing on a structure with five main themes without sub-themes. The proposal was sent back and forth to make sure there was consensus among the authors about the final thematic structure.

### Reflexivity statement

All the authors were clinical psychologists who have worked with adolescents in therapy and participated actively in the data collection. We, therefore, had our own preconceptions about adolescents coming reluctantly to therapy, and the services they are offered. Therefore, reflexivity and working actively to keep an open and explorative attitude towards the phenomena under study were key elements of the entire research process [37]. Hence, it was particularly important that there were two moderators whenever possible and the whole team actively participated in the analytic process.

### Ethics

Participation in the focus groups was voluntary and all participants gave their informed consent after being informed about their right to withdraw at any time. However, because the focus groups were organized through the heads of the clinics, some of the participants might have felt pressure to attend them. Participants could choose how actively they wanted to engage in group discussions during the focus groups. Because we interviewed existing teams, we knew we entered a sphere with established power structures and team culture. We were therefore very aware to any signs of discomfort, and questions were constructed open and explorative to maximize participants’ reflections and experiences and minimalize chances of participants feeling that their participation could discredit them in front of their leaders. As expected, some participants took the floor more, while others chose to speak less. There was no indication of participants feeling pressured to participate. Given the design, facilitators encouraged minimal participation and invited less active participants in, but did not push towards participation.

The participants were working with adolescents in a vulnerable situation and were, at the same time, asked to share information about how they saw and dealt with adolescents in the context of their work. Despite being instructed not to share identifiable third-party information, there was an ethical consideration whether it would be possible to recognize adolescents from concrete examples. Therefore, we actively attended to the participants’ descriptions of their adolescent clients when collecting the data and writing the article.

## Findings

It became clear while analyzing the material that different perspectives about adolescent clients coexisted between workplaces, between coworkers within the same workplace, and even within the individual therapists, depending on the adolescent they had in mind when talking. This seemed to represent both individually based attitudes related to the clinical tasks and clinical populations, and shared subcultural or organizational understandings, where different teams had developed a language and a way to talk about the work they were doing. Two teams in the same location had even developed rather distinct ways to talk about the work they were doing and the persons they met through their work. Our analysis resulted in five themes describing different sets of understandings when dealing with an adolescent who came to therapy at someone else’s initiative: *The hurt and distrustful adolescent; The adolescent lacking hope for the future; The adolescent engulfed in the burden of mental*-*health suffering; The adolescent as something more than a psychiatric patient; and The adolescent meeting a system with varying flexibility and space for engagement.*

### The hurt and distrustful adolescent

Many of the participants emphasized how upbringing and early relationships affect the adolescent clients’ basis for trusting their therapist. Adolescents exist in a larger social system, consisting of families, school, and friends. In all the teams, some of the therapists reflected on how these larger systems could explain adolescents’ resistance to therapy, and how they could undermine good treatment outcomes. In some teams, this perspective was prominent. A couple of participants described how an adolescent’s reluctance to be in treatment made sense when the system around the adolescent was not on par:

But I think that it is basic. If the kid does not thrive at home, or in the institution, and has not yet come into foster care, or if there is marginal support in the school or there is bullying, or psychological or physical violence at home, it makes no difference coming here once a week to talk to me or to you.

The participants expressed the understanding that many of the adolescent clients they meet have been let down by family, school, or institutions, and therefore, they find it difficult to trust another adult. Furthermore, many adolescent clients are still living in families that have problems (e.g., a parent with a psychiatric illness, alcohol or drug addiction, violence and/or abuse) and it might be scary, or even dangerous, to talk about this:

There are also a lot of kids who have had to adapt to the adults, right? Adults that have not met them [kids] in a good way; therefore, they [the kids] have found a way. It can be a lot of serious stuff going on at home. […] They are living it. They live with a very ill father or an alcoholic mother or what not, and then we almost expect that they should want to open up while they live it. So, I have great understanding for [that]. … I would not have liked that others had said that I, at any moment, should be ready to talk about that which was the most difficult thing going on in my life, when it suited them; right?

### The adolescent lacking hope for the future

Many participants across teams understood adolescents’ lack of engagement in treatment as a sign of a more general lack of faith in their own power to change their life into something better:

Yes, it’s not certain that they, eh, if they don’t have the experience of being ok, then they won’t necessarily have an idea, or I don’t expect that they believe it can be ok. It is just bad. There is no trust that someone magically can make it good. Or “not you,” kind of “What can you do?” It is hopeless. I’ve had it like this for ever, as long as I have been alive.

Participants had also observed that some adolescents had previous experience attending treatment sessions for a long time without experiencing any positive changes, and even having negative experiences. They, therefore, had no trust that things would be different this time. Such experiences could lead to lack of motivation, which was reflected in the adolescents’ resistance to therapy:

I do have a patient who doesn’t want to come because she has had a very unfortunate experience with the outpatient clinic she belongs to, right? And a lot of things happened over her head which were misinterpreted and such, yeah, so she was having a very, aversion… it seems like a scary experience which makes her, I don’t know if it is about being afraid of saying something and then suddenly something happens that she didn’t mean, so yeah.

### The adolescent engulfed in the burden of mental-health suffering

Therapists in some of the teams discussed the way the burden of mental-health problems, in themselves, can actually be the reason for the reluctance observed in adolescent clients. They exemplified how, for example, strong anxiety, eating disorders, or psychosis could interfere in different ways with treatment participation by affecting how adolescents viewed themselves and their surroundings: “At the same time, different mental disorders, in themselves, affect how we understand ourselves and the world, which complicates it even more, right?”

Some participants also talked about the way we can see something as reluctance when it really is a part of the illness and the underlying reason the person needs therapy:

I also think that some of them have major discomfort, serious anxiety issues, and that they might not understand how they can get help; it is just so unimaginably unpleasant to come here, so they don’t manage it.

The therapist here expresses an understanding where aspects of mental health problems, like avoidance or lack of hope, in themselves can act as a barrier to treatment, thus resulting in avoidance of therapy.

### The adolescent as something more than a psychiatric patient

In all the teams, the therapists discussed adolescent clients’ lack of motivation and symptoms at length. Yet, individual therapists in all the teams also raised complementary perspectives, describing how other aspects of the clients´ lives than their mental illness were important, and the significance of paying attention to their life outside of therapy. They emphasized that adolescents also have strengths, abilities, and interests, and this theme was related to the way therapists conceptualized and understood the adolescent’s problems. Many therapists expressed a genuine interest and joy in working with adolescents:

Participant 1: And they share so much of this [leisure activities] willingly, it is totally like… I know nothing, about these things. But, being curious, it is amazing how… how they appreciate it. I don’t believe that they…, a lot of them here are not used to adults being interested in their leisure interests.

Facilitator: So, you describe very clearly such a … clear interest and support in the adolescents…

Participant 2: We are interested in it [many laugh]. They [adolescents] are so amazing! There are a lot of us that especially like to work with adolescents.

Participants also talked about the importance of trusting the strengths of adolescents; i.e., that they are capable of making choices on their own, and that they have the capacity to work through things. The participants in several interviews communicated their view that adolescents are resourceful, whether at school, or in sports, or finding ways to handle their situation. Since many of the adolescents have been through a lot, they necessarily must have found ways of coping with life. Sometimes their ways of coping later became the problems they were referred for. One of the participants explained how she understood some of these problems:

Well, they [adolescents] have their ways of solving problems. Like you say. But to see it as something…Well, it is basically a resource to try and solve things. To frame it like it is… but to develop some alternative ways [to solve things], which one at least… If one only has one way of solving a problem, then one does not have a choice. It is good to have choices. So one can at least build…

The fact that this participant referred to the identified problematic behavior of adolescent client as a form of problem-solving, made it something the therapist could use in therapy to help the adolescent find alternative methods of coping.

Participants in most of the interviews also mentioned at least once how clients’ interests and lives could be a resource in therapy. They described how they enjoyed getting to know the adolescents and learning about their interests, and they emphasized how this connection enhanced the therapeutic relationship:

When we talk about things that are not fun, then it quickly can become like “oh”, they [the adolescents] freeze a bit and won’t do anything. But, if we talk about their interests in a session, then you suddenly see a different person, and something comes forth, which might be useful to know.

### The adolescent meeting a system with varying flexibility and space for engagement

In addition to the described differences in how therapists individually or as teams conceptualized why adolescents could come to therapy reluctantly, different treatment teams also varied in the way they described the adolescent’s, and consequently the therapists’, role in treatment. While some teams organized their services based on an understanding of the adolescents as a suffering person in need of care, others viewed the adolescent as a customer of a product (healthcare). Although the welfare system ensures that the political and economic basis for outpatient clinics are equally distributed, different subcultures emerged in how different treatment teams, even in the same clinic, conceptualized their tasks and the corresponding roles of adolescents in treatment. Client roles seemed to be ascribed to the adolescent client partly based on the preconceptions of clinicians, and partly based on the leader’s and the treatment team’s understanding of the expectations of the healthcare system and its organization of services.

At the flexible end of the clinic continuum, therapists experienced a large degree of responsibility for getting through to the adolescents and they adjusted their work-days and the organization of services to meet the perceived needs of the adolescents and the support systems around the adolescents. They described days that consisted of car rides or longer work-days to be more available to adolescent clients:

We are like an outpatient clinic on wheels. We have, I don t know, I’m talking for myself, I have set days out in the municipalities where I have most [adolescents]. Like, almost every Friday I am in X, and then the first-line services know I’m there, they can ask for consultations if they have things they want to discuss. Or we call them in for sessions.

The core managerial values in other treatment teams seemed to be effectiveness and case-load responsibility. Teams in these clinics typically put less emphasis on tailoring individual services and had a strong focus on efficient use of resources and the assessment of who would benefit from treatment. The metaphor of the customer was often used to describe a situation in which treatment was warranted, typically described as a situation where the client had sufficient faith in the product (treatment) for treatment efforts to proceed. They discussed problems concerning lack of motivation when someone else (e.g., the parents) was the customer and the adolescent had no self-interest in being there:

No, … they sit together with, often their mother, who then tells a lot and turns to the kid and asks the kid to confirm; the kid says little or nothing, or looks at the watch or asks: “Can we leave now?” Then, it is very clear that I do not have a customer relationship for what we are doing, but a pretty eager caregiver wanting the kid to get help, but instead it becomes a communication which does not add up.

Therapists in teams with a strong focus on the customer metaphor were also more active in bringing pressure to bear on mental-health care services and prioritization when considering if, and when, the system should adapt the product to be desirable for the customer or motivate the customer to buy into what they can offer:

But I believe that within that frame we spoke of before that we have become more like: “Who am I doing this for?” It has something to do with the frame and we think we are going to use a lot of time on those who, who do not want to, if we think that we won’t make it in a way within reasonable time, or shall we call it a day and instead…help those who want to, maybe.

Participants in different treatment teams also described large differences in the flexibility they experienced within the organization in which they were working. This affected the way they thought about motivation and the clients’ role in the system, as well as their experience of doing the tasks they were expected to do. While some groups did not express a need to adapt, other therapists felt some organizational frameworks stopped them from prioritizing consistently with their clinical assessment, resulting in the most vulnerable adolescents who needed time to open up not always getting this time because of the way the services were organized. A participant working in one of the specialized teams described the flexibility and their experience with how this affected the way they could work with adolescents with severe problems but low motivation:

Facilitator: Yes. So, you have flexibility considering that too.

Participant 1: Yes, that was what I thought to say, that we take walks with them or they can have a car ride with us, or we can come home.

Participant 2: Sometimes. And just practically, things they want to achieve. Go to the store or a café or get to activities which are important to carry out. We are lucky, like that. We have a broad spectrum of things we can do. And that makes me think that maybe I find it easier working with kids who do not want to come here in this job than in other workplaces where the frames are narrower in terms of what one can offer.

A participant in a different treatment team described how the organizational framework of the treatment team also provided a guide as to when flexibility within the framework was warranted due to the severity of the problem, and when lack of motivation was an indication of ill-timing of treatment or a lack of need for treatment:

It has to do with the framework we have. They too, in a way, say something about what the severity must be in order for us to, in a way, prioritize use of time on that problem. […] If the problems are big enough then they will work it out within the framework and if not, then maybe the problems are not as big after all.

Thus, this theme shed light on the variety of services adolescents meet, even within the framework of a strong welfare state, and consequently, the variety of work contexts in which therapists treat adolescents who come to therapy at another person’s initiative.

## Discussion

One of the most striking findings of the present study was the difference in conceptualization and organization of services that emerged between and within clinics. These differences were prominent in different teams in terms of both the language used to describe adolescent clients and staff’s understanding of their responsibility and needed flexibility of services, i.e., more or less flexibility to adapt to the person in question. We were surprised how different subcultures seemed to emerge between the clinics, and even between teams in the same clinic, even in the context of a strong welfare system and with rather clear regulations of services. All clinics included in the study are part of public mental-health services, thus operate within the same regulations, and with the same economic incentives. It became clear, however, that different teams interpreted consequences of regulation for service provision quite differently. Implications of this will be discussed below.

In addition to unexpectedly large differences between treatment teams, there were more expected variation and differences in how individual therapists perceived, met, and talked about their adolescent clients. This variation is natural, and may be expected, unavoidable and even wanted, because individual therapists bring so much more than professional knowledge and skills to their clinical work. The observed variation does, however, have implications for leaders, as they point to the significance of leaders being aware of the variation and coexistence of perspectives among therapists so these can be explored and utilized to the best for services and clients. The findings also point to the importance of establishing team cultures where there is room for expressing and exploring different perspectives and understandings of adolescent clients and therapist roles and responsibility when meeting adolescent clients.

How, then, can we understand the large variation between treatment teams’ conceptualizations and organization of services? And what implications does this variation have? The study was carried out in the context of a strong welfare system. Within a welfare system, equal opportunities and the distribution of goods are important principles. That is, one wants equivalent services and the same access to services, irrespective of social class, geography, or which therapist a client happens to meet. In order to achieve this, the Norwegian government has over the past two decades introduced strong political guidelines and New Public Management (NPM) as a control system to manage costs and the distribution of goods and services. This has resulted in greater similarity in what services are offered within the mental health care system, regardless of geographical location. It has, however, also resulted in large reorganizations of how clinics report activities, where therapists now are expected to report all activity in pre-determined categories of activities. Further, an important question rising from our findings is how control systems like NPM, which introduces its own terminology and shape activity, influences clinical thinking, language, and practices. Terms such as “customer,” “productivity,” “prioritizing,” and “patient flow” are at the heart of NPM. We were surprised to observe that while some teams kept their terminology from their clinical training when discussing and reflecting on adolescent clients, other teams had adopted the terminology of NPM. They referred to their clients as customers, and concepts of patient flow and productivity took precedence over the first-person perspective of the adolescent when considering service organization and clinical decisions.

The coexistence of different language traditions between treatment teams has important implications, as language, and especially biased language, can affect attitudes [38, 39]. The organization, management, and provision of healthcare services are, therefore, likely to influence both the therapists’ perceived clinical responsibilities and tasks, and the reluctant adolescents’ motivation to come and stay in therapy. Our findings thus point to important and relevant questions in an era where the healthcare sector is increasingly influenced by control systems that were developed in very different contexts. This highlights the significance of understanding what therapists do to build alliances with adolescents who come reluctantly to therapy. Adolescents’ experience coming to treatment at another’s initiative also point to the need for future research to explore whether adoption of this new language is affecting clinical thinking and rationale for interventions, and thus change clinical practice. In many ways, then, the variation observed at the level of healthcare services can be seen as extensions of dilemmas linked to variations in therapist behaviors observed in clinical encounters. While some variation is needed and wanted, there is a need to reduce unwanted variance. Critics of increased external control systems in mental health services, like NPM, do for example emphasize the need for flexibility to adapt to the individual client [40]. Returning to psychotherapy research, it has been shown, for example, that “appropriate responsiveness,” or a therapist’s ability to adapt therapeutic behavior to cues from the interpersonal environment is crucial for outcomes [41]. Moreover, Owen and Hilsenroth [42] found that even variability within the course of therapy is associated with better outcomes. Adolescents themselves emphasize the importance of individual adjustments, flexibility, and creativity in a therapeutic session [16] and enough flexibility to fit with their way of life [43]. However, some therapists are not able to help their clients and may cause harm [44]; e.g., 14–24% of adolescents have negative outcomes from psychotherapy [45]. These lines of research stress the importance of variation and flexibility to achieve good outcomes in therapy, while clearly showing that variation in itself is not necessarily positive. How, then, can one know what is wanted variation and what is unwanted variation in services? And what happens with respect to flexibility and variability in clinical work when control systems, like NPM, are introduced into a clinical setting?

Our findings are also relevant to the literature on therapist effects. Research on adult clients has, for example, shown that a therapist’s interpersonal skills, such as the ability to convey empathy [44] and give a convincing rationale for clinical activities [46] are important for outcomes, with more effective therapists being able to form strong alliances across a range of clients [47, 48]. Such interpersonal factors are considered to be especially important when working with adolescents [19]. It is very important for adolescents to meet a friendly and kind therapist who cares and is a genuine person [16, 43]. The therapist also needs to be nonjudgmental and open regarding adolescents experiences, and clearly convey their tolerance [49]. On the other hand, a therapist who is not caring or acts superior could ruin both an adolescent’s faith in psychotherapy and their ability to trust other adults in the future [16].

The substantial variation found in this study, indicate that the treatment adolescents gets and the degree of flexibility clinicians provide vary substantially—both across and within clinics. This is problematic in light of the goal of equivalent services, and point to the significance of team leaders and therapists increasing their awareness of, and finding ways to explore and discuss, differences in therapist perspectives within and across clinics. Such processes could be one way to develop services to reduce unwanted variation and develop ways to utilize natural variation between therapists beneficially. Further research is still needed to explore whether the observed variations in attitudes are affecting therapist behavior and effect of treatment, and to determine what is wanted, and what is unwanted, variation in services.

## Strengths and limitations

This study provides descriptions of how therapists view and conceptualize an important group of adolescent clients: i.e., those who come to therapy at the initiative of others. This is clearly an important and understudied population, and a basic function of qualitative research is to examine such gaps in knowledge. As such, we consider this exploratory aim to be an important strength of the present study. At the same time, we acknowledge several limitations that need to be taken into account when planning future research. First, the exploratory design of this study did not allow us to differentiate wanted from unwanted variation in our findings. This will be important to explore in future research. Second, all the participants in our study were working in the same healthcare context, which may decrease the value of our findings for therapists in different contexts. Third, while therapists are important stakeholders in performing clinical interventions, there is also a need to explore the first-person perspectives of individuals who are in therapy at the initiative of others. Fourth, while focus-group methodology has advantages for exploring situations that are common among participants, divergent views may be more difficult to obtain because of group dynamics. An example is participants holding back information in fear of negatively affecting collegial relationships. This may be particularly so in this study, as we chose to include the leaders of the same employee groups as participants. Finally, using a different analytical approach, such as discourse analysis, may have enabled an in-depth analysis of the multiple meanings in the language that therapists use when describing adolescents struggling with mental-health issues.

## Conclusion

We studied the perspectives of 51 therapists about adolescents who enter mental-health treatment at the initiative of others. Based on a thematic analysis of ten focus groups with these participants, we extracted five broad themes: *The hurt and distrustful adolescent; The adolescent lacking hope for the future; The adolescent engulfed in the burden of mental*-*health suffering; The adolescent as something more than a psychiatric patient;* and *The adolescent meeting a system with varying flexibility and space for engagement.* The observed differences in how therapists perceive, meet, and talk about the adolescents they work with and the services adolescents are offered, point to the complexity of the landscape that therapists navigate, balancing the needs of adolescents against perceived clinical responsibility, flexibility, and tasks.

## Data Availability

The datasets used and/or analyzed during the current study are available from the corresponding author on reasonable request.

## Abbreviation

NPM: New Public Management
